# Does Preoperative Waiting Time Affect the Short-Term Outcomes and Prognosis of Colorectal Cancer Patients? A Retrospective Study from the West of China

**DOI:** 10.1155/2022/8235736

**Published:** 2022-04-30

**Authors:** Xiao-Yu Liu, Zi-Wei Li, Bing Kang, Yu-Xi Cheng, Wei Tao, Bin Zhang, Hua Zhang, Zheng-Qiang Wei, Dong Peng

**Affiliations:** ^1^Department of Gastrointestinal Surgery, The First Affiliated Hospital of Chongqing Medical University, Chongqing 400016, China; ^2^Department of Clinical Nutrition, The First Affiliated Hospital of Chongqing Medical University, Chongqing 400016, China

## Abstract

**Purpose:**

The purpose of this study is to analyze the effect of preoperative waiting time on the short-term outcomes and prognosis in colorectal cancer (CRC) patients.

**Methods:**

We retrospectively analyzed 3744 CRC patients who underwent primary CRC surgery at a single clinical medical center from Jan 2011 to Jan 2020. The baseline information, short-term outcomes, overall survival (OS), and disease-free survival (DFS) were compared among the short-waiting group, the intermediate-waiting group, and the long-waiting group.

**Results:**

A total of 3744 eligible CRC patients were enrolled for analysis. There were no significant differences in all of the baseline information and short-term outcomes among the three groups. In multivariate analysis, older age (OS: *p*=0.000, HR = 1.947, 95% CI = 1.631–2.324; DFS: *p*=0.000, HR = 1.693, 95% CI = 1.445–1.983), advanced clinical stage (OS: *p*=0.000, HR = 1.301, 95% CI = 1.161–1.457; DFS: *p*=0.000, HR = 1.262, 95% CI = 1.139–1.400), overall complications (OS: *p*=0.000, HR = 1.613, 95% CI = 1.303–1.895; DFS: *p*=0.000, HR = 1.560, 95% CI = 1.312–1.855), and major complications (OS: *p*=0.001, HR = 1.812, 95% CI = 1.338–2.945; DFS: *p*=0.006, HR = 1.647, 95% CI = 1.153–2.352) were independent factors of OS and DFS. In addition, no significant difference was found in all stages (OS, *p*=0.203; DFS, *p*=0.108), stage I (OS, *p*=0.419; DFS, *p*=0.579), stage II (OS, *p*=0.465; DFS, *p*=0.385), or stage III (OS, *p*=0.539; DFS, *p*=0.259) in terms of OS and DFS among the three groups.

**Conclusion:**

Preoperative waiting time did not affect the short-term outcomes or prognosis in CRC patients.

## 1. Introduction

Colorectal cancer (CRC) is the third most common cancer and one of the leading causes of cancer-related death globally [1]. Radical surgery is the curable treatment for resectable CRC patients, and patients with metastatic CRC are typically offered chemotherapy (fluoropyrimidines plus either oxaliplatin or irinotecan) and might also receive biological drugs targeting VEGF (bevacizumab) and if they have RAS wild-type tumors, EGFR (cetuximab or panitumumab) [2–5]. The outcomes and prognosis of CRC patients after surgery are associated with age, type 2 diabetes mellitus (T2DM), tumor stage, and postoperative complications [6–8].

Endoscopy/CT/MRI/fecal occult blood test (FOBT) is recommended for the initial detection of CRC and the result of colonoscopic biopsy is the gold standard for confirmed CRC cases [9]. These examinations might be delayed by medical facility, excessive load of the center hospital, and patients' and their families' hesitation about surgery [10]. Unfortunately, the association between diagnostic, therapeutic delays and prognosis in CRC patients were unclear.

Some studies reported that the longer waiting time was not the risk factor of worse outcomes in CRC patients [11–13]. However, Pita-Fernández et al. concluded that short diagnostic intervals were significantly associated with higher mortality in rectal cancers, and longer diagnostic intervals were not associated with poorer prognosis in CRC patients [14]. Thus, the purpose of this study is to analyze the effect of preoperative waiting time on the short-term outcomes and prognosis in CRC patients.

## 2. Materials and Methods

### 2.1. Patients

We retrospectively collected 5473 CRC patients who underwent primary CRC surgery at a single clinical medical center from Jan 2011 to Jan 2020. This study was conducted following the World Medical Association Declaration of Helsinki. We obtained the ethical approval from the Institutional Ethics Committee of the First Affiliated Hospital of Chongqing Medical University (2021-520), and all the patients signed informed consents.

### 2.2. Inclusion and Exclusion Criteria

The eligible patients for this study were selected by the following criteria. The exclusion criteria were as follows: 1, incomplete medical records of patients (*n* = 761); 2, stage IV CRC (*n* = 875); 3, non-R0 resection (*n* = 25); and 4, neoadjuvant chemotherapy (*n* = 68). Finally, a total of 3744 CRC patients were included in this study.

### 2.3. Surgery Management and Patients' Follow-Up

According to the clinical guideline of AJCC 8^th^ Edition, [15] the surgeons performed the radical CRC surgery for all the patients included in this study. The records of patients' follow-up were obtained through the outpatient system and telephone interviews.

### 2.4. Definitions

The clinical stage of patients was in accordance with the guideline of AJCC 8^th^ Edition [15]. The waiting time was defined as the time from the suspect examination of colonoscopy/CT/MRI/FOBT to CRC surgery. The short-waiting group included the patients with the waiting time less than a month, the intermediate-waiting group included the patients with the waiting time between one month to two months, and patients with the waiting time more than two months were defined as the long-waiting group. The postoperative complications were defined according to the Clavien-Dindo classification, and ≥III grade complications were considered as major complications [16]. Overall survival (OS) was defined as the time from CRC surgery to last follow-up or death. Disease-free survival (DFS) was calculated from CRC surgery to the recurrence of primary tumor, last follow-up, or death.

### 2.5. Data Collection

We retrospectively collected the baseline information and the short-term outcomes through electronic medical records system. Data on age, sex, body mass index (BMI), type 2 diabetes mellitus (T2DM), smoking, drinking, hypertension, laparoscopy, family history, tumor location, and clinical stage were collected as the baseline information. The short-term outcomes included operation time, blood loss, retrieved lymph nodes, hospital stay, overall complications, and major complications.

### 2.6. Statistical Analysis

Continuous variables were expressed as the mean ± SD, and the Kruskal-Wallis test was used to compare the differences among the three groups. Categorical variables were expressed as *n* (%), and chi-square test was used for comparison. The Kaplan-Meier test was performed to compare the different clinical stages of CRC patients on OS and DFS, and Cox regression analyses were conducted to identify independent predictive factors for OS and DFS. We used SPSS (version 22.0) statistical software for data analysis. A bilateral *p* value of <0.05 was considered statistically significant.

## 3. Results

### 3.1. Clinical Characteristics of the Patients

**Figure 1:**
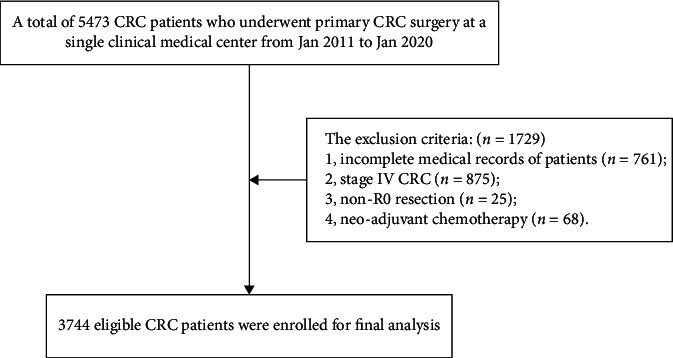
Flow chart of patient selection.

A total of 5473 patients were identified in the clinical medical center database. According to the inclusion and exclusion criteria, we finally enrolled 3744 eligible CRC patients for analysis, which was shown in Figure 1. The patients were divided into three groups in this study, including 2533 patients in short-waiting group, 845 patients in intermediate-waiting group, and 366 patients in long-waiting group. After pooling up all of the data, there were no significant differences in all of the baseline information among the three groups, including age, sex, BMI, T2DM, smoking, drinking, hypertension, laparoscopy, family history, tumor location, or clinical stage (*p* > 0.05) (Table 1).

### 3.2. Short-Term Outcomes

We compared the short-term outcomes among the three groups, and it was found that no differences in operation time (*p*=0.126), blood loss (*p*=0.054), retrieved lymph nodes (*p*=0.288), hospital stay (*p*=0.183), overall complications (*p*=0.412), or major complications (*p*=0.881) (Table 2).

### 3.3. Univariate and Multivariate Analysis of OS/DFS

The medium follow-up time was 31 (1–113) months. In univariate analysis, age (*p*=0.000, HR = 2.105, 95% CI = 1.770–2.504), BMI (*p*=0.011, HR = 0.948, 95% CI = 0.923–0.974), T2DM (*p*=0.009, HR = 1.380, 95% CI = 1.085–1.754), clinical stage (*p*=0.000, HR = 1.255, 95% CI = 1.121–1.404), overall complications (*p*=0.000, HR = 1.893, 95% CI = 1.590–2.253), and major complications (*p*=0.000, HR = 2.855, 95% CI = 2.027–4.021) were risk factors of OS. In multivariate analysis, age (*p*=0.000, HR = 1.947, 95% CI = 1.631–2.324), BMI (*p*=0.036, HR = 0.802, 95% CI = 0.678–0.950), clinical stage (*p*=0.000, HR = 1.301, 95% CI = 1.161–1.457), overall complications (*p*=0.000, HR = 1.613, 95% CI = 1.303–1.895), and major complications (*p*=0.001, HR = 1.812, 95% CI = 1.338–2.945) were independent factors of OS (Table 3).

In terms of DFS, in univariate analysis, age (*p*=0.000, HR = 1.774, 95% CI = 1.517–2.075), clinical stage (*p*=0.000, HR = 1.226, 95% CI = 1.106–1.358), overall complications (*p*=0.000, HR = 1.762, 95% CI = 1.498–2.071), and major complications (*p*=0.000, HR = 2.471, 95% CI = 1.765–3.458) were significant risk factors. In multivariate analysis, age (*p*=0.000, HR = 1.693, 95% CI = 1.445–1.983), clinical stage (*p*=0.000, HR = 1.262, 95% CI = 1.139–1.400), overall complications (*p*=0.000, HR = 1.560, 95% CI = 1.312–1.855), and major complications (*p*=0.006, HR = 1.647, 95% CI = 1.153–2.352) were independent factors as well (Table 4).

### 3.4. Prognosis in Different Stages

**Figure 2:**
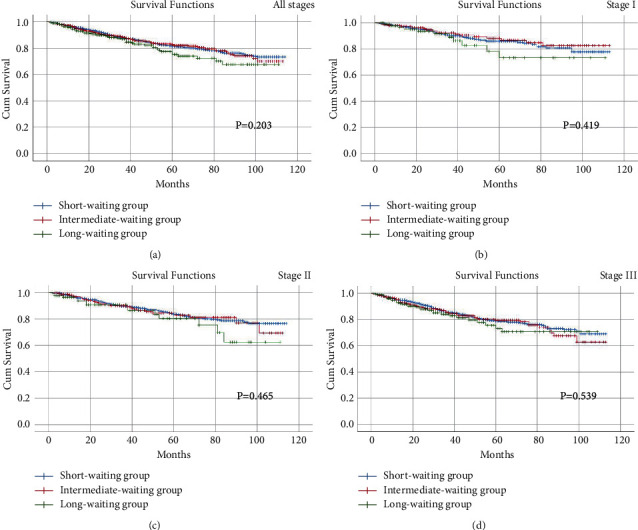
Overall survival among three groups, including short-waiting group, intermediate-waiting group, and long-waiting group. (a) stage I; (b) stage II; (c) stage III; (d) stage IV.

**Figure 3:**
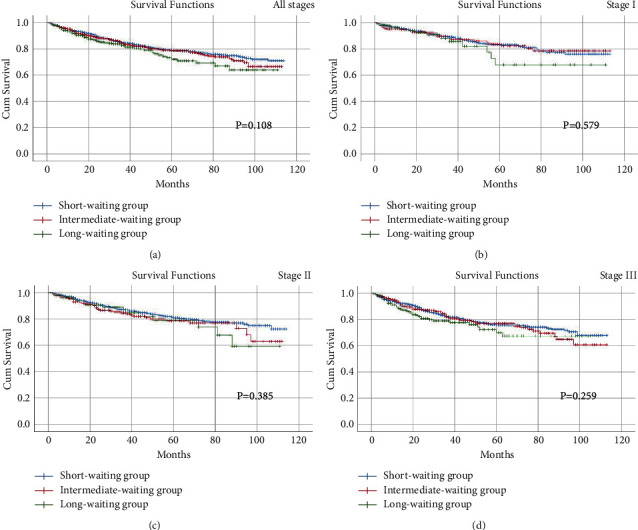
Disease-free survival among three groups, including short-waiting group, intermediate-waiting group, and long-waiting group. (a) stage I; (b) stage II; (c) stage III; (d) stage IV.

The Kaplan-Meier curve was conducted to analyze the prognosis on different stages of CRC patients. As a result, no differences were found in all stages (OS, *p*=0.203; DFS, *p*=0.108), stage I (OS, *p*=0.419; DFS, *p*=0.579), stage II (OS, *p*=0.465; DFS, *p*=0.385), or stage III (OS, *p*=0.539; DFS, *p*=0.259) in terms of OS and DFS among the three groups (Figures 2 and 3).

## 4. Discussion

A total of 3744 eligible CRC patients were included for analysis. There were no significant differences in all of the baseline information and short-term outcomes among the three groups. In multivariate analysis, older age, advanced clinical stage, overall complications, and major complications were independent factors of OS and DFS. In addition, no significant difference was found in all stages, stage I, stage II, or stage III in terms of OS and DFS among the three groups.

Early stage CRC was recommended for radical CRC surgery. However, for advanced CRC, tumors grow and evolve through a constant crosstalk with the surrounding microenvironment, and emerging evidence indicates that angiogenesis and immunosuppression frequently occur simultaneously in response to this crosstalk. Accordingly, strategies combining antiangiogenic therapy and immunotherapy seem to have the potential to tip the balance of the tumor microenvironment and improve treatment response [2, 4, 5, 17]. Furthermore, immunocheckpoint and cytotoxic T lymphocyte antigen-4 (CTLA-4) is an inhibitory immune checkpoint that can be expressed in tumor-infiltrating lymphocytes and colorectal cancer (CRC) cells [3]. Therefore, chemotherapy, antiangiogenic therapy, and immunotherapy were effective treatments.

In this study, we focused on the clinical I–III stage CRC patients. Excessive waiting time for cancer selective surgery was received with concern [18, 19]. It was demonstrated that preoperative waiting time might have influence on the oncological surgery [20]. The variation in waiting time might increase the psychological stress on patients, but provide more sufficient preoperative reservation of organic function especially in elderly patients, leading to the differences in surgical outcomes of breast cancer and prostate cancer [21–23]. Peng et al. reported that the longer preoperative waiting time of gastric cancer patients contributed to the shorter postoperative hospital stay; however, longer waiting time had no impact on OS [24]. In terms of CRC, some studies reported that preoperative waiting time was not associated with postoperative outcomes and survival [25–27]. However, Pita-Fernández et al. drew an inverse conclusion that shorter waiting time was connected with higher mortality in rectal cancer patients [14]. Thus, we conducted this study to analyze the effect on waiting time in CRC patients.

Short-term outcomes tend to reflect the postoperative situation directly. A few studies reported that no association was found between delayed diagnosis and short-term outcomes in CRC patients [12]. In accordance with previous conclusions, this study found that waiting time was not associated with short-term outcomes. The factor mostly affecting the outcomes was still tumor staging [28] Individuals awaiting evaluation for the surgery experienced an increased level of anxiety. However, CRC is a slow-growing disease, and longer waiting time had no significant adverse effect on the outcomes [25, 29].

The prognosis of CRC patients was currently concentrated by surgeons. It was revealed that the OS and DFS of CRC patients were affected by age, underlying diseases, tumor staging, and postoperative complications, which was largely consistent with our conclusions [30, 31]. Some previous studies did not find an association between the waiting time and survival [11–13]. Similarly, our results showed that preoperative waiting time in CRC patients was not an independent risk factor for OS or DFS. Furthermore, the prognosis on different tumor stages of CRC was analyzed, respectively, and we found that OS and DFS were not affected by preoperative waiting time in different tumor stages. The exact mechanism was unclear. It was deduced that preoperative waiting time was relatively short during the process of malignancy development, thus it might not exactly affect the survival [11]. Besides, the waiting time could be well utilized for preparation of cardio-pulmonary function before surgery, which was preponderant for survival, and unfortunately, some other factors than diagnostic delay might have more effect on prognosis.

There were some strengths in our study. First, to our knowledge, this study included the largest amount of data evaluating the preoperative waiting time on the outcomes of CRC patients, which could make the results more reliable. Second, the short-term outcomes were compared among the three groups in this study, which were not reported previously. Third, we firstly analyzed the prognosis with different tumor stages (stage I, stage II, and stage III) among the three groups.

However, some existing limitations were mentioned necessarily in this study. First, this was a single-center retrospective study in the west of China, which might cause selection bias. Second, the median follow-up time was relatively short. Third, the information including neoadjuvant therapy and postoperative therapy was lacking. Fourth, stage IV CRC patients were excluded, these patients would receive biological drugs targeting therapy or immune therapy, and they might be also affected by preoperative waiting time. Thus, multicenter prospective randomized controlled trials with comprehensive information and all stages of CRC should be performed in the future.

In conclusion, preoperative waiting time did not affect the short-term outcomes or prognosis in CRC patients.

## Figures and Tables

**Table 1:** Baseline information of the different waiting groups.

Characteristics	Short-waiting group (*n* = 2533)	Intermediate-waiting group (*n* = 845)	Long-waiting group (*n* = 366)	*p* value
Age (year)	62.8 ± 11.9	63.6 ± 11.8	63.8 ± 12.6	0.072
Sex				0.549
Male	1458 (57.6%)	504 (59.6%)	210 (57.4%)	
Female	1075 (42.4%)	341 (40.4%)	156 (42.6%)	
BMI (kg/m^2^)	22.7 ± 3.2	22.8 ± 3.3	22.9 ± 3.1	0.492
T2DM	282 (11.1%)	107 (12.7%)	55 (15.0%)	0.070
Smoking	946 (37.3%)	332 (39.3%)	129 (35.2%)	0.375
Drinking	768 (30.3%)	270 (32.0%)	106 (29.0%)	0.527
Hypertension	641 (25.3%)	228 (27.0%)	114 (31.1%)	0.051
Laparoscopy	2206 (87.1%)	749 (88.6%)	326 (89.1%)	0.337
Family history	77 (3.0%)	29 (3.4%)	15 (4.1%)	0.526
Tumor location				0.334
Colon	1192 (47.1%)	382 (45.2%)	159 (43.4%)	
Rectum	1341 (52.9%)	463 (54.8%)	207 (56.6%)	
Clinical stage				0.086
I	493 (19.5%)	190 (22.5%)	91 (24.9%)	
II	672 (26.5%)	214 (25.3%)	86 (23.5%)	
III	1368 (54.0%)	441 (52.2%)	189 (51.6%)	

*Note.* Variables are expressed as the mean ± SD, *n* (%); ^*∗*^*p* value <0.05. T2DM, type 2 diabetes mellitus; BMI, body mass index.

**Table 2:** Short-term outcomes of the different waiting groups.

Characteristics	Short-waiting group (2533)	Intermediate-waiting group (845)	Long-waiting group (366)	*p* value
Operation time (min)	227.3 ± 79.7	224.2 ± 87.0	229.5 ± 86.9	0.126
Blood loss (mL)	105.7 ± 154.7	99.5 ± 133.3	99.4 ± 124.3	0.054
Retrieved lymph nodes	15.0 ± 7.6	15.2 ± 7.8	15.4 ± 7.3	0.288
Hospital stay (day)	11.7 ± 9.5	11.1 ± 6.5	11.1 ± 6.7	0.183
Overall complications	556 (22.0%)	204 (24.1%)	81 (22.1%)	0.412
Major complications	65 (2.6%)	20 (2.4%)	8 (2.2%)	0.881

*Note.* Variables are expressed as the mean ± SD, *n* (%); ^*∗*^*p* value <0.05.

**Table 3:** Univariate and multivariate analysis of overall survival.

Risk factors	Univariate analysis		Multivariate analysis	
	HR (95% CI)	*p* value	HR (95% CI)	*p* value
Age (>/≤64, years)	2.105 (1.770–2.504)	0.000^*∗*^	1.947 (1.63–2.324)	0.000^*∗*^
Sex (female/male)	0.924 (0.779–1.096)	0.363		
BMI (>/≤22.6)	0.948 (0.923–0.974)	0.011^*∗*^	0.802 (0.678–0.950)	0.036^*∗*^
Hypertension (yes/no)	1.062 (0.878–1.286)	0.535		
T2DM (yes/no)	1.380 (1.085–1.754)	0.009^*∗*^	1.169 (0.91–1.491)	0.207
Tumor site (colon/rectum)	1.119 (0.946–1.324)	0.191		
Clinical stage (III/II/I)	1.255 (1.121–1.404)	0.000^*∗*^	1.301 (1.161–1.457)	0.000^*∗*^
Smoking (yes/no)	1.058 (0.891–1.256)	0.522		
Drinking (yes/no)	1.072 (0.895–1.284)	0.451		
Family history (yes/no)	0.680 (0.400–1.156)	0.154		
Waiting time (long/intermediate/short)	1.099 (0.967–1.250)	0.148		
Overall complications (yes/no)	1.893 (1.590–2.253)	0.000^*∗*^	1.613 (1.303–1.895)	0.000^*∗*^
Major complications (yes/no)	2.855 (2.027–4.021)	0.000^*∗*^	1.812 (1.338–2.945)	0.001^*∗*^

*Note*. ^*∗*^*p* value <0.05. HR, hazard ratio; CI, confidence interval; BMI, body mass index; T2DM, type 2 diabetes mellitus.

**Table 4:** Univariate and multivariate analysis of disease-free survival.

Risk factors	Univariate analysis		Multivariate analysis	
	HR (95% CI)	*p* value	HR (95% CI)	*p* value
Age (>/≤64, years)	1.774 (1.517–2.075)	0.000^*∗*^	1.693 (1.445–1.983)	0.000^*∗*^
Sex (female/male)	0.919 (0.785–1.075)	0.289		
BMI (>/≤22.6)	0.864 (0.741–1.009)	0.065		
Hypertension (yes/no)	1.054 (0.885–1.256)	0.553		
T2DM (yes/no)	1.196 (0.951–1.504)	0.127		
Tumor site (colon/rectum)	1.036 (0.888–1.210)	0.650		
Clinical stage (III/II/I)	1.226 (1.106–1.358)	0.000^*∗*^	1.262 (1.139–1.400)	0.000^*∗*^
Smoking (yes/no)	1.065 (0.909–1.246)	0.436		
Drinking (yes/no)	1.096 (0.930–1.293)	0.274		
Family history (yes/no)	0.655 (0.399–1.075)	0.094		
Waiting time (long/intermediate/short)	1.004 (0.998–1.011)	0.204		
Overall complications (yes/no)	1.762 (1.498–2.071)	0.000^*∗*^	1.560 (1.312–1.855)	0.000^*∗*^
Major complications (yes/no)	2.471 (1.765–3.458)	0.000^*∗*^	1.647 (1.153–2.352)	0.006^*∗*^

*Note*. ^*∗*^*p* value <0.05. HR, hazard ratio; CI, confidence interval; BMI, body mass index; T2DM, type 2 diabetes mellitus.

## Data Availability

The datasets used and analyzed during the current study are available from the corresponding author on reasonable request.
